# Phosphorylation of human enhancer filamentation 1 (HEF1) stimulates interaction with Polo-like kinase 1 leading to HEF1 localization to focal adhesions

**DOI:** 10.1074/jbc.M117.802587

**Published:** 2017-11-30

**Authors:** Kyung Ho Lee, Jeong-Ah Hwang, Sun-Ok Kim, Jung Hee Kim, Sang Chul Shin, Eunice EunKyeong Kim, Kyung S. Lee, Kunsoo Rhee, Byeong Hwa Jeon, Jeong Kyu Bang, Hyunjoo Cha-Molstad, Nak-Kyun Soung, Jae-Hyuk Jang, Sung-Kyun Ko, Hee Gu Lee, Jong Seog Ahn, Yong Tae Kwon, Bo Yeon Kim

**Affiliations:** From the ‡ World Class Institute, Anticancer Agent Research Center, Korea Research Institute of Bioscience and Biotechnology, 30 Yeongudanji-ro, Ochang, Cheongwon, Chungbuk 28116, Korea,; the ¶ Biomedical Research Institute, Korea Institute of Science and Technology, Hwarangno 14-gil 5, Seongbuk-gu, Seoul 02792, Korea,; the ‖ Laboratory of Metabolism, Center for Cancer Research, National Cancer Institute, Bethesda, Maryland 20892,; the § Department of Biological Sciences, Seoul National University, Seoul 08826, Korea,; the ** Research Institute of Medical Sciences, Department of Physiology, College of Medicine, Chungnam National University, Daejeon 35015, Korea,; the ‡‡ Division of Magnetic Resonance, Korea Basic Science Institute, Ochang 28119, Korea,; the §§ Genome Structure Research Center, Korea Research Institute of Bioscience and Biotechnology, 125 Gwahak-ro, Yuseong-gu, Daejeon 34141, Korea, and; the ¶¶ Protein Metabolism Medical Research Center and Department of Biomedical Sciences, College of Medicine, Seoul National University, Seoul 03080, Korea

**Keywords:** cell migration, focal adhesion, phosphorylation, protein targeting, protein-protein interaction, proton transport, serine/threonine protein kinase, Plk1, HEF1-Plk1 complex, HEF1 translocation, HEF1 pSer-780, HEF1 pThr-804

## Abstract

Elevated expression of human enhancer filamentation 1 (HEF1; also known as NEDD9 or Cas-L) is an essential stimulus for the metastatic process of various solid tumors. This process requires HEF1 localization to focal adhesions (FAs). Although the association of HEF1 with FAs is considered to play a role in cancer cell migration, the mechanism targeting HEF1 to FAs remains unclear. Moreover, up-regulation of Polo-like kinase 1 (Plk1) positively correlates with human cancer metastasis, yet how Plk1 deregulation promotes metastasis remains elusive. Here, we report that casein kinase 1δ (CK1δ) phosphorylates HEF1 at Ser-780 and Thr-804 and that these phosphorylation events promote a physical interaction between Plk1 and HEF1. We found that this interaction is critical for HEF1 translocation to FAs and for inducing migration of HeLa cells. Plk1-docking phosphoepitopes were mapped/confirmed in HEF1 by various methods, including X-ray crystallography, and mutated for functional analysis in HeLa cells. In summary, our results reveal the role of a phosphorylation-dependent HEF1–Plk1 complex in HEF1 translocation to FAs to induce cell migration. Our findings provide critical mechanistic insights into the HEF1–Plk1 complex–dependent localization of HEF1 to FAs underlying the metastatic process and may therefore contribute to the development of new cancer therapies.

## Introduction

Human enhancer filamentation 1 (HEF1, also known as Cas-L or NEDD9) belongs to the Cas scaffold protein family (Efs/Sin and p130Cas/Bcar), which mediates signal transduction through protein-protein interactions (1, 2). HEF1 is present at the focal adhesion (FA),^5^ where cells attach to the extracellular matrix in an integrin-dependent manner, and is phosphorylated by focal adhesion kinase (FAK) (1). The elevated expression of HEF1 has been identified as an essential stimulus for cancer cell metastasis (3–5). Although FA-associated HEF1 is thought to play an important role in cancer cell migration (1, 6), the mechanism by which HEF1 is targeted to FAs remains poorly understood.

Polo-like kinase 1 (Plk1) is a well-known mitotic kinase and has been widely reported as a key player in cell division, centrosome maturation, and bipolar spindle formation during multiple stages of mitosis (7–9). By contrast, the function of non-mitotic Plk1 has received little attention. Plk1 contains an N-terminal catalytic domain and a substrate-binding C-terminal Polo box domain (PBD). The PBD of Plk1 forms a complex through the conserved phospho-Ser/Thr (p-Ser/p-Thr)-binding module of its substrate (10, 11). Strong positive correlations between human cancer metastasis and the up-regulation of Plk1 have been reported (12–14); however, the mechanism by which Plk1 deregulation promotes metastasis and tumorigenesis remains largely unknown.

Casein kinase 1 (CK1) is a family of serine/threonine kinases. To date, six human (α, δ, ϵ, γ_1_, γ_2_, and γ_3_) isoforms and one bovine (β) isoform have been identified (15, 16). Among them, both CK1δ and CK1ϵ are considered important regulators of oncogenesis, which comes from the abnormal regulation of either the Wnt or the Hedgehog signaling pathway (15, 17). Despite the high amino acid identity in their catalytic domains (97% identity), CK1δ and CK1ϵ have been reported to exhibit distinct functions (18, 19). However, the functional differences between CK1δ and CK1ϵ have only recently been explored.

The FA is a multiprotein complex that contains a large number of proteins, such as paxillin, talin, integrin, α-actinin, vinculin, and FAK (20, 21). FAs usually serve as the connection between the actin cytoskeleton and extracellular matrix. Therefore, cell migration is accompanied by FA disassembly. Disassembly of FAs is an acutely regulated cellular process (22–24). Recently, Rho- and Rac-independent, microtubule-induced FA disassembly has been reported to be mediated by the FAK and dynamin (24). However, the exact molecular mechanism by which the FA-docking protein is translocated into the FA site has not yet been well characterized.

An indirect relationship between Plk1 and HEF1 in the HEF1 degradation machinery has been suggested (19). However, the presence of a direct relationship between these two proteins is not known. In this study, we identified a novel phosphorylation-dependent HEF1 transportation mechanism that is directly regulated by Plk1. We demonstrated that CK1δ phosphorylates HEF1 at Ser-780 and Thr-804 residues, which allows non-mitotic Plk1 to dock to one of the resulting two phosphoepitopes. Furthermore, the docking of non-mitotic Plk1 on HEF1 was shown to be critical for HEF1 to translocate to FAs and for cell migration in HeLa cells.

## Results

### Phosphorylation-dependent interaction between the C-terminal region of HEF1 and Plk1

Our previous attempts to identify novel Plk1-binding partners and novel Plk1 functions in primary cilium disassembly suggested that Plk1 has an indirect effect on HEF1 during HEF1 degradation (19). We therefore attempted here to elucidate the functional relationship between Plk1 and HEF1 in cellular processes. To this end, we first closely monitored the physical interaction between HEF1 and Plk1. We demonstrated an interaction, direct or indirect, between HEF1 and Plk1 during cell cycle progression using an immunoprecipitation assay, with thymidine-treated, asynchronously growing, or nocodazole-arrested 293T cells.

**Figure 1.**
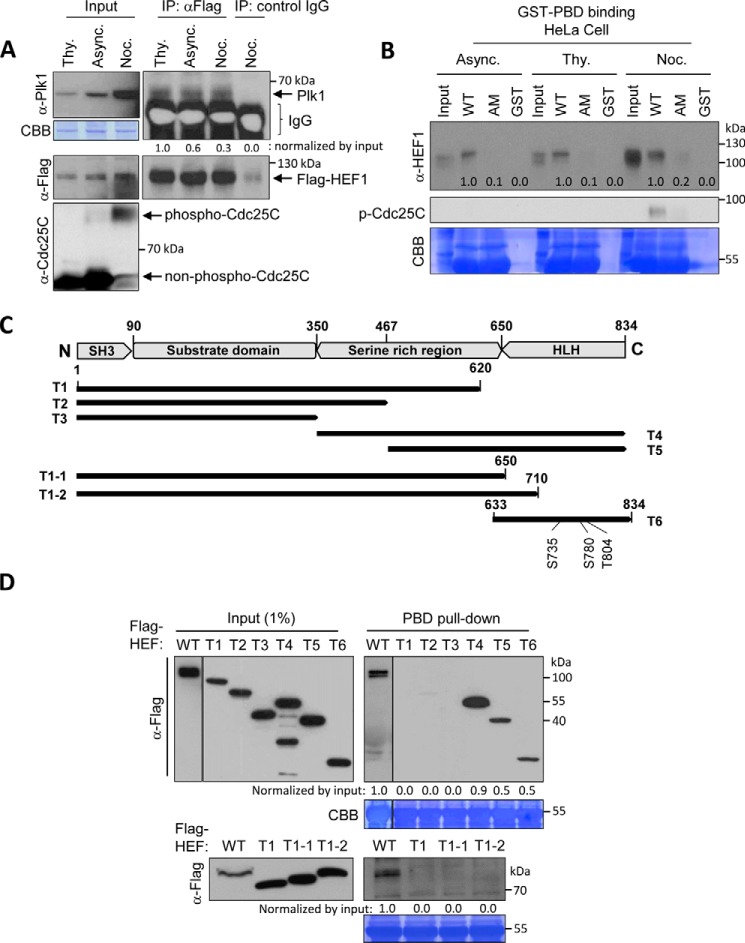
**C-terminal region of HEF1 interacts with Plk1 PBD in a phosphorylation-dependent manner.** *A*, HEF1 forms a complex with Plk1 throughout the cell cycle. The lysates of FLAG-HEF1–transfected HeLa cells were prepared under thymidine (*Thy*)-treated (S phase), asynchronously growing (*Async*), or nocodazole (*Noc*)-treated (M phase) conditions. The lysates were immunoprecipitated with anti-FLAG antibody and then immunoblotted with the indicated antibodies. *B*, HEF1 interacts with Plk1 PBD in a phosphorylation-dependent manner. The total cell lysates were prepared from HeLa cells in three different cell stages, as indicated in *A*, and then pulled down with bead-conjugated GST-PBD WT, GST-PBD AM (H538A/K540M) (11), or control GST only. The precipitates were immunoblotted with an anti-HEF1 antibody. Note that the endogenous HEF1 efficiently bound to GST-PBD WT, but not to the AM. CBB represents the amount of loaded protein. *C*, schematic of the domain structure and truncation mutants of HEF1 used in this study. Amino acid numbers and domain names are indicated. *D*, C-terminal region of HEF1 interacts with Plk1 PBD. The wild-type, full-length HEF1 (*WT*) and each of the truncated mutants of HEF1 were transfected into HEK293T cells. The resulting cell lysates were subjected to PBD pulldown assays using GST-Plk1 PBD WT, and membranes were then immunoblotted with an anti-FLAG antibody. CBB represents the amount of GST-Plk1 PBD WT. The immunoblots shown were obtained from one of three independent experiments and are representative of the overall results. Band intensities were quantified with ImageJ and normalized as indicated in the figure, and the relative values are shown *below* the bands.

HEF1 continuously formed a complex with Plk1 in each cell cycle population (Fig. 1*A*). Additionally, throughout the cell cycle, HEF1 mainly bound to the WT PBD rather than to AM (a phospho-dependent binding-defective mutant; PBD H538A/K540M mutant) (11) (Fig. 1*B*). The continual binding of HEF1 to Plk1 during cell cycle progression may reflect the HEF1 function present in all cell cycle stages in combination with Plk1.

Next, to identify the phosphorylation site on HEF1 responsible for Plk1 PBD binding, we generated eight truncation mutant constructs of HEF1 based on its domain structure (Fig. 1*C*) and performed a PBD pulldown assay using these mutants. Among the eight deletion mutants, three C-terminal regions (T4, T5, and T6) bound to Plk1 PBD, whereas the N-terminal regions (T1, T2, T3, T1-1, and T1-2) did not (Fig. 1*D*). Thus, the region of HEF1 comprising aa 710–834 was identified as a minimal region containing the Plk1 PBD-binding phosphorylation sites. Taken together, these results show that the HEF1 aa 710–834 region binds to Plk1 PBD in a phosphorylation-dependent manner, and it may work together with Plk1 throughout the entire cell cycle.

### HEF1 forms a complex with Plk1 PBD through pSer-780 and pThr-804 epitopes on HEF1

**Figure 2.**
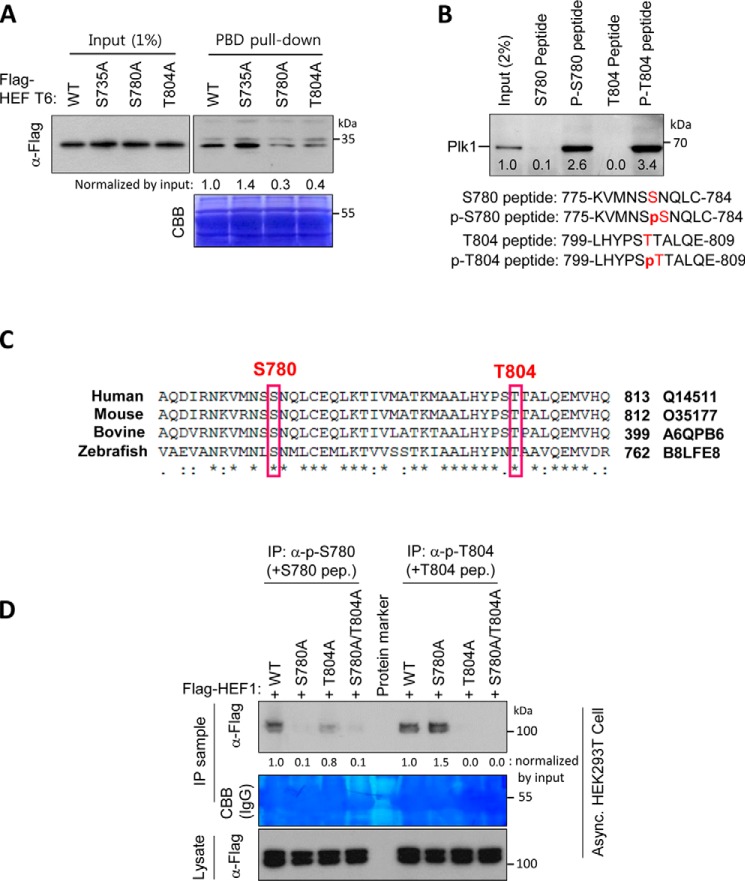
**HEF1 forms a complex with Plk1 PBD through pSer-780 and pThr-804 epitopes on HEF1.** *A*, mutation of either S780A or T804A disrupted the HEF1–Plk1 PBD interaction. Either the FLAG-tagged HEF1 T6-WT or each of the alanine substitution mutants (Ser → Ala or Thr → Ala) was transfected into HEK293T cells. The resulting cell lysates were subjected to PBD pulldown assays using GST-Plk1 PBD WT, and then membranes were immunoblotted with an anti-FLAG antibody. CBB represents the amount of loaded GST-Plk1 PBD WT protein. *Numbers* in *A* indicate relative amounts of HEF1 bound to GST-PBD. *B*, HEF1 pSer-780 and pThr-804 peptides interact with endogenous Plk1. HeLa cell lysates were incubated with bead-conjugated HEF1 non-phospho-Ser-780, phospho-Ser-780, non-phospho-Thr-804, or phospho-Thr-804 peptides. The resulting precipitates were subjected to an immunoblotting analysis with an anti-Plk1 antibody. The HEF1-derived peptide sequences are shown in the immunoblot *below. C*, HEF1 Ser-780 and Thr-804 residues are conserved across the species. Shown is amino acid sequence alignment of the C-terminal region of HEF1 among different species. Conserved Ser-780 and Thr-804 residues are marked as *red boxes* from fish to humans. Amino acid numbers and accession numbers are indicated. *D*, phosphorylation-specific antibodies generated against pSer-780 and pThr-804 epitopes on HEF1 were confirmed by IP-immunoblot analysis. The FLAG-tagged HEF1 WT, S780A, T804A, or S780A/T804A double mutant was transfected into HEK293T cells. Cell lysates were immunoprecipitated with either an anti-phospho-Ser-780 or -Thr-804 antiserum, with 5 μg/ml of the non-phospho-Ser-780 (+*S780 pep*) or non-phospho-Thr-804 (+*T804 pep*) peptide, and then immunoblotted with an anti-FLAG antibody. The immunoblots shown were obtained from one of three independent experiments and are representative of the overall results. Band intensities were quantified with ImageJ and normalized as indicated in the figure, and the relative values are shown *below* the bands.

We then chose three candidate sites (Ser-735, Ser-780, and Thr-804) from the sequence in the HEF1 aa 710–834 region, based on the consensus Plk1 PBD-binding module ((φ/P)φ(T/Q/H/M)S(pS/pT)(P/*X*), where φ is a hydrophobic residue) (11). After selection by visual scanning, either a Ser → Ala or a Thr → Ala substitution mutant for each candidate site was generated on the HEF1-T6 construct. Subsequent PBD-binding assays revealed that the S780A or T804A mutation severely hindered the HEF1–Plk1 PBD binding, whereas the S735A mutation did not do so (Fig. 2*A*). Consistent with this, in a peptide-binding assay, only the phospho-Ser-780 and phospho-Thr-804 peptides interacted with endogenous Plk1, whereas their respective non-phosphorylated peptides did not (Fig. 2*B*). In line with the importance of pSer-780 and pThr-804 epitopes to PBD binding, both of these residues were conserved across various species (Fig. 2*C*), and a mass spectrometry analysis revealed the presence of both pSer-780 and pThr-804 peptides in thymidine-treated HeLa cells (Table 1 and Fig. S1*A*). Their phosphorylation *in vivo* was further confirmed in immunoprecipitation (IP)-immunoblotting analyses using phospho-specific antibodies, which were generated against either the pSer-780 or the pThr-804 epitope (Fig. 2*D*). As expected, HEF1 WT was immunoprecipitated by both phospho-antisera, whereas S780A- or T804A-containing HEF1 mutant was not immunoprecipitated by anti-pSer-780 or anti-pThr-804 antiserum, respectively.

**Table 1:**
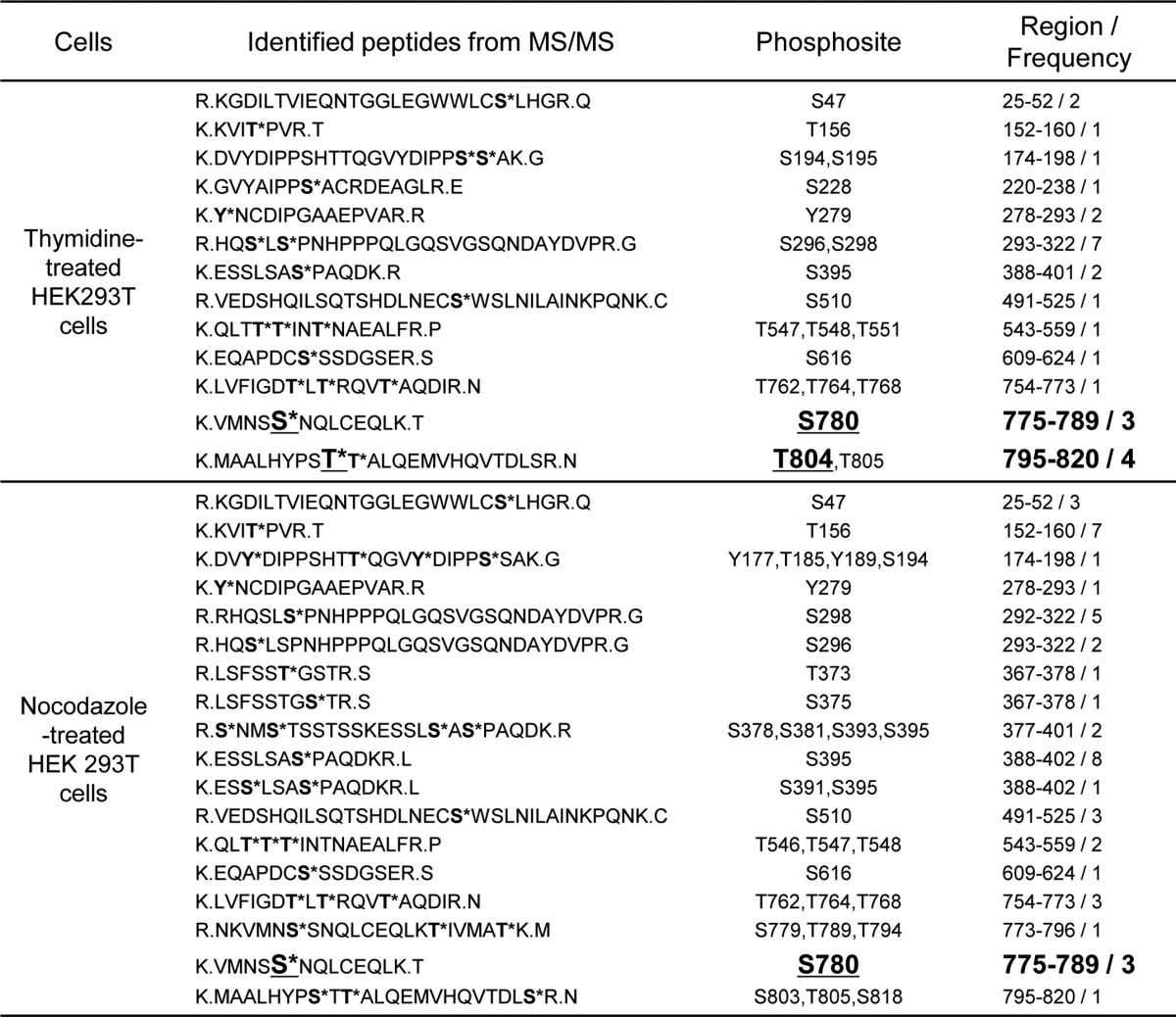
**Phosphopeptides from HEF1 immunoprecipitates identified by mass spectrometry analysis**

Asterisks indicate phosphorylated residues. Underlines indicate the pSer-780 and pThr-804 residues in thymidine-/nocodazole-treated HEK293T cells. Identified phosphopeptides from HEF1 IP-MS analyses and frequencies of phosphopeptide retrieval from each sample are shown. The larger-sized letters were used to highlight the pSer-780 and pThr-804 peptides.

### Structural analysis of the Plk1 PBD and phospho-HEF1 peptide complex

**Figure 3.**
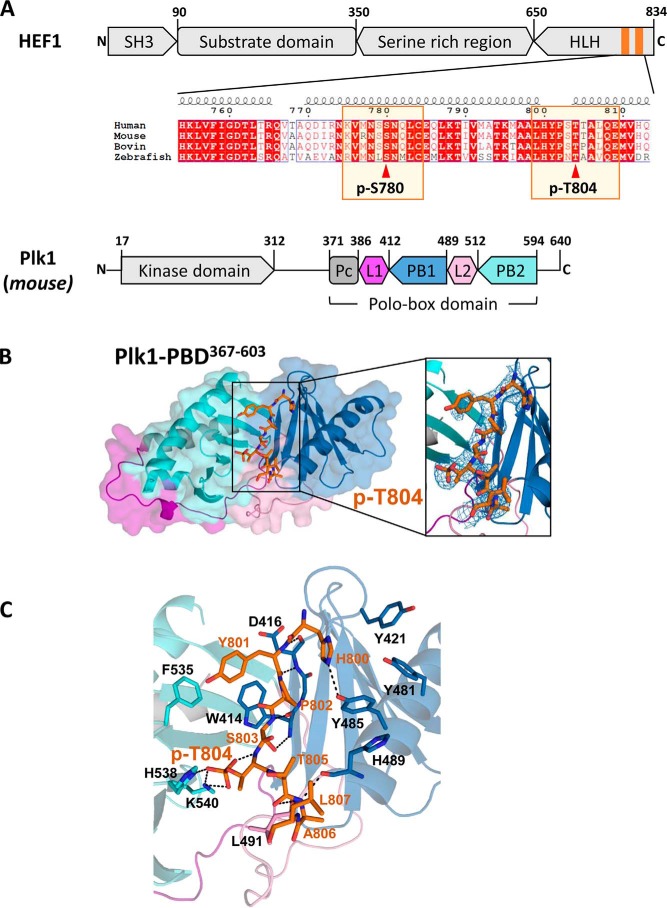
**Crystal structure of the Plk1 PBD and phospho-HEF1 peptide complex.** *A*, *schematic* of domain structures of HEF1 and Plk1. Amino acid numbers and domain names are indicated. PBD of Plk1 consists of Polo cap (*Pc*), linker 1 (*L1*), Polo box 2 (*PB1*), linker 2 (L2) and Polo box 2 (*PB2*). Shown is sequence alignment of HEF1 for the two phosphosites (indicated by *arrowheads*) with the secondary structure depicted *above* the sequence. *B*, overall structure of the Plk1 PBD and pThr-804 peptide complex. Plk1 PBD is shown in a *surface presentation* following the same *color scheme* as in *A*. The pThr-804 peptide is shown in an *orange stick model* with the electron density map (omit map) contoured at the 1.2 σ level. *C*, interactions between Plk1 PBD and pThr-804 HEF1 peptide. Only the key residues of Plk1 are shown for clarity, and hydrogen bonds are indicated by *dashed lines*. The PDB accession number is 5X3S.

To verify the phospho-dependent interactions between Plk1 PBD and HEF1, we attempted to determine the complex structures of the phosphopeptides of HEF1 with Plk1 PBD (Fig. 3*A*). Two phosphorylated peptides corresponding to ^775^KVMNSpSNQLC^784^ and ^799^LHYPSpTTALQE^809^ of HEF1, referred to as pSer-780 and pThr-804 peptides, respectively, were synthesized. Each peptide was complexed with Plk1 PBD, and crystallization attempts were made. However, only the Plk1 PBD complexed with pThr-804 peptide gave diffraction quality crystals (see Table 2 and “Experimental procedures” for details). The final model included all Plk1 PBD (residues 371–593) and ^800^HYPSpTTAL^807^ of the pThr-804 peptide because the terminal residues were not clearly defined in the electron density maps. There were two copies of the complex in the asymmetric unit of the crystal, which were almost identical, with a root mean square deviation value of 0.36 Å for the 208 Cα atoms.

**Table 2:** **Statistics on data collection and refinement**

	Plk1-PBD and pThr-804 complex
Beam line	PAL 5C
Wavelength (Å)	0.9795
Space group	P2_1_

**Unit cell parameters**	
*a* (Å)	57.564
*b* (Å)	59.440
*c* (Å)	72.748
α (degrees)	90
β (degrees)	99.48
γ (degrees)	90
Resolution range (Å)	50–2.9 (3.0–2.9)*^a^*
No. of total/unique reflections	477,131/10,907
Completeness (%)	84.4 (67.3)
*I*/σ(*I*)	7.6 (1.7)
*R*_merge_*^b^* (%)	15.2 (27.2)
Resolution range (Å)	50–2.9
*R*-value/*R*_free_*^c^* (%)	20.3/24.4
No. of protein atoms	3746
No. of water molecules	7

**Root mean square deviation from ideal geometry**	
Bond lengths (Å)	0.004
Bond angles (degrees)	0.662

**Average *B*-factor (Å^2^)**	38.7
Protein	38.9
pThr-804	32.4
Water molecules	30.6

**Ramachandran analysis (%)**	
Favored	94.7
Allowed	5.3
Outliers	0

*^a^* Values in parentheses are for the outermost resolution shell.

*^b^ R*_merge_ = Σ*_h_*Σ*_i_*|*I*(*h*,*i*) − 〈*I*(*h*)〉|/Σ*_h_*Σ*_i_ I*(*h*,*i*), where *I*(*h*,*i*) is the intensity of the *i*th measurement of reflection *h* and 〈*I*(*h*)〉 is the mean value of *I*(*h*,*i*) for all *i* measurements.

*^c^ R*_free_ was calculated from a randomly selected 5% (450 reflections) set of reflections not included in the calculation of the *R* value.

The crystal structure determined at 2.9 Å resolution shows that the pThr-804 peptide is bound in a groove formed by Polo box 1 (*PB1*; consisting of residues 412–489) and Polo box 2 (*PB2*; consisting of residues 512–594) of Plk1 PBD (Fig. 3, *B* and *C*). The core of the pThr-804 peptide adopts a β-structure, forming four hydrogen bonds with the last strand of the β-sheet in Polo box 1 of Plk1. In particular, Tyr-801 and Ser-803 of the pThr-804 peptide form hydrogen bonds with the backbone amides and carbonyls of Asp-416 and Trp-414 of Plk1, respectively. The phosphate moiety of the pThr-804 peptide binds to the positively charged binding pocket, forming hydrogen bonds with the side chains of Lys-540 and His-538 of Plk1, and stabilizes the backbone conformation of the peptide by forming hydrogen bonds with the backbone amide of Thr-804. In addition, the carbonyl of Ser-803 forms hydrogen bonds with the backbone amide of Thr-805, stabilizing the conformation. The imidazole ring of His-800 of the pThr-804 peptide is located at the tyrosine cluster of Plk1 PBD and forms hydrogen bonds with Tyr-485, whereas Tyr-801 is on the hydrophobic binding surface formed by Trp-414 and Phe-535 of Plk1. There are additional interactions between the C-terminal region of the peptide and the L2 region of Plk1 PBD. The interface between Plk1 PBD and the pThr-804 peptide is estimated as ∼490 Å per polypeptide, and the detailed interactions are shown in Fig. 3*C* (PDB code 5X3S). Interestingly, the key residues involved in the interactions seem to be highly conserved. The overall binding mode for the phosphopeptide of HEF1 to Plk1 is quite similar to that reported previously (*i.e.* the interactions with the β-sheet of Polo box 1 and the interactions around the phosphothreonine are almost the same as in the previous report), whereas the interactions seen for the terminal ends of the peptide seem to vary somewhat, depending on the sequence (25, 26). Therefore, the structural analysis clearly suggests that the phosphothreonine at position 804 of HEF1 (pThr-804) plays an important role in specific interaction and recognition by Plk1 PBD.

### CK1δ generates pSer-780 and pThr-804 epitopes on HEF1 and induces the formation of the HEF1–Plk1 complex

**Figure 4.**
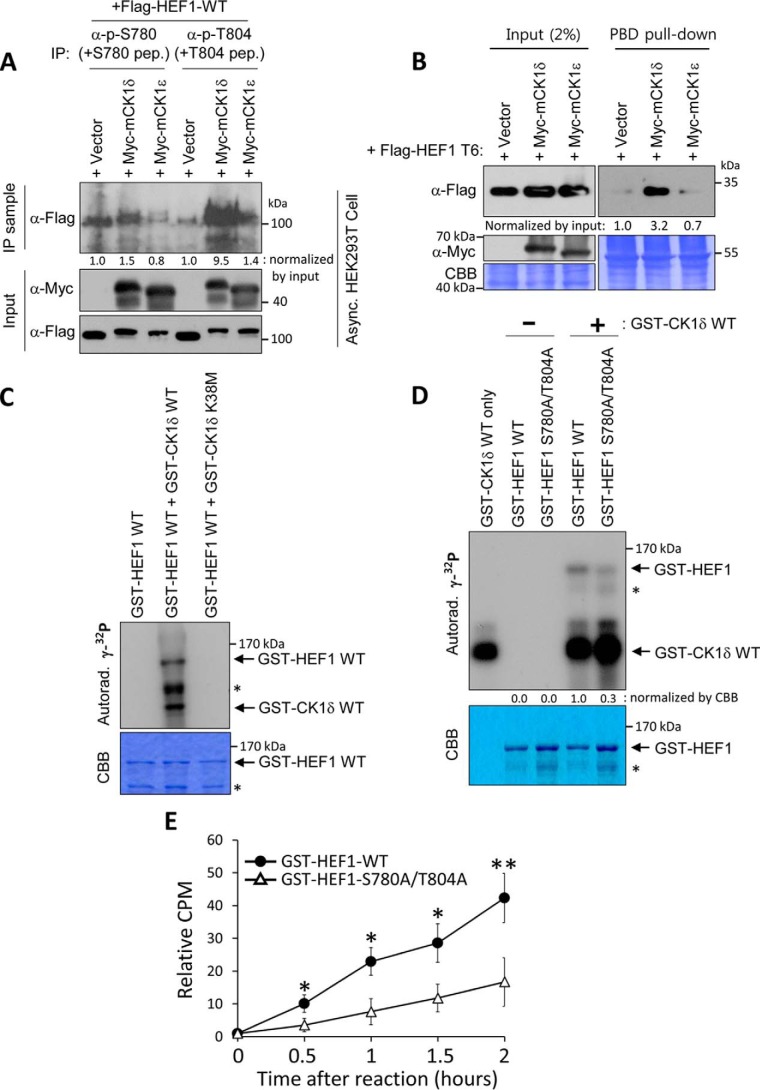
**CK1δ induces the phosphorylation of Ser-780 and Thr-804 residues on HEF1, leading to the formation of the HEF1–Plk1 complex.** *A*, expression of CK1δ induces the phosphorylation of Ser-780 and Thr-804 residues on HEF1. HEK293T cells co-transfected with FLAG-HEF1 WT and a Myc-empty vector (+*Vector*), Myc-tagged CK1δ (+*Myc-CK1*δ) vector, or CK1ϵ (+*Myc-CK1*ϵ) vector were subjected to an immunoprecipitation assay. Cell lysates were immunoprecipitated with either anti-phospho-Ser-780 or -Thr-804 antiserum, with 10 μg/ml non-phospho-Ser-780 (+*S780 pep*) or non-phospho-Thr-804 (+*T804 pep*) peptide, respectively, and then immunoblotted with an anti-FLAG antibody. Cell lysates were probed with either anti-FLAG or -Myc antibody. *B*, expression of CK1δ induces HEF1–Plk1 complex formation. HEK293T cells co-transfected with FLAG-HEF1 T6 and a Myc-empty vector (+*Vector*), Myc-tagged CK1δ (+*Myc-CK1*δ) vector, or CK1ϵ (+*Myc-CK1*ϵ) vector were subjected to a PBD pulldown assay using GST-Plk1 PBD WT. The resulting precipitates were immunoblotted with the indicated antibodies. Note that the expression of CK1δ greatly enhances FLAG-HEF1 T6 binding to Plk1 PBD. *C*, CK1δ directly phosphorylates HEF1. The bacterially purified GST-HEF1 WT proteins were reacted with either a bacterially purified GST-CK1δ WT or K38M (kinase-dead mutant) in the presence of [γ-^32^p]ATP, and the resulting samples were then separated by 10% SDS-PAGE and exposed on an X-ray film (Autorad). CBB represents the amount of loaded protein. *Asterisks* indicate degradation products of GST-HEF1 protein. *D*, HEF1 S780A/T804A double mutant reduces HEF1 phosphorylation by CK1δ. The bacterially purified GST-CK1δ WT proteins were reacted with either a bacterially purified GST-HEF1 WT or S780A/T804A mutant in the presence of [γ-^32^p]ATP, and the resulting samples were then separated by 10% SDS-PAGE and exposed on an X-ray film (Autorad). CBB represents the amount of loaded protein. *Asterisks* indicate degradation products of GST-HEF1 protein. *E*, time course of HEF1 WT and HEF1 S780A/T804A mutant phosphorylation by CK1δ. The kinase reaction was carried out as indicated in *D*. A dried SDS-polyacrylamide gel band was excised and dissolved in 30% H_2_O_2_. Phosphorylation (cpm) was measured by liquid scintillation counting. **, *p* < 0.01; *, *p* < 0.05 (unpaired two-tailed *t* test). *Error bars*, S.D. from three independent experiments. Data were normalized against the amount of loaded protein that the CBB showed. The immunoblots shown were obtained from one of three independent experiments and are representative of the overall results. Band intensities were quantified with ImageJ and normalized as indicated in the figure, and the relative values are shown *below* the bands.

In accordance with our previous finding that CK1δ and CK1ϵ phosphorylate the priming phosphorylation sites on Dvl2 for Plk1 PBD binding (19), it could be assumed that both of these kinases also act as the pSer-780– and pThr-804–generating kinases on HEF1. Accordingly, we observed that the expression of CK1δ efficiently induced pSer-780 and pThr-804 epitopes in a kinase overexpression experiment, whereas, by comparison with CK1δ, CK1ϵ changed these phosphoepitopes to a lesser degree (Fig. 4*A*). In line with these observations, clear enhancement of Plk1 PBD binding to FLAG-HEF1 T6 was observed in the CK1δ-overexpressing sample. On the other hand, altering CK1ϵ did not result in substantial changes in this experiment (Fig. 4*B*). Thus, we surmised that, although CK1δ and CK1ϵ share high similarity at the amino acid level, there may be limited functional redundancy between them in the generation of pSer-780 and pThr-804 epitopes on HEF1. We also observed the direct phosphorylation of HEF1 by CK1δ in bacterially purified forms of GST-HEF1 and GST-CK1δ (Fig. 4*C*). Strong HEF1 phosphorylation signals were observed in the CK1δ WT-incubated samples, whereas HEF1, alone or in combination with a CK1δ kinase-inactive mutant form (K38M), did not produce any specific signal (Fig. 4*C*). In line with these observations, a clear reduction in CK1δ-generated phosphorylation of HEF1 was observed by S780A/T804A double mutation of HEF1 (Fig. 4*D*). The HEF1 S780A/T804A double mutant remarkably reduced HEF1 phosphorylation by CK1δ in comparison with that for HEF1 WT in an *in vitro* kinase assay. In addition, in the time course experiments of HEF1 WT and HEF1 S780A/T804A phosphorylation by CK1δ, the difference in the extent of phosphorylation between HEF1 WT and S780A/T804A was 2.8-fold after 30 min, 3-fold after 1 h, 2.5-fold after 1.5 h, and 2.5-fold after 2 h. The difference in phosphorylation between the two was statistically significant over time (Fig. 4*E*). These results strongly support the idea that the activity of CK1δ is important for the phosphorylation of both the Ser-780 and Thr-804 residues of HEF1, resulting in the formation of the HEF1–Plk1 complex.

### Formation of the HEF1–Plk1 complex is essential for HEF1 localization to FAs

**Figure 5.**
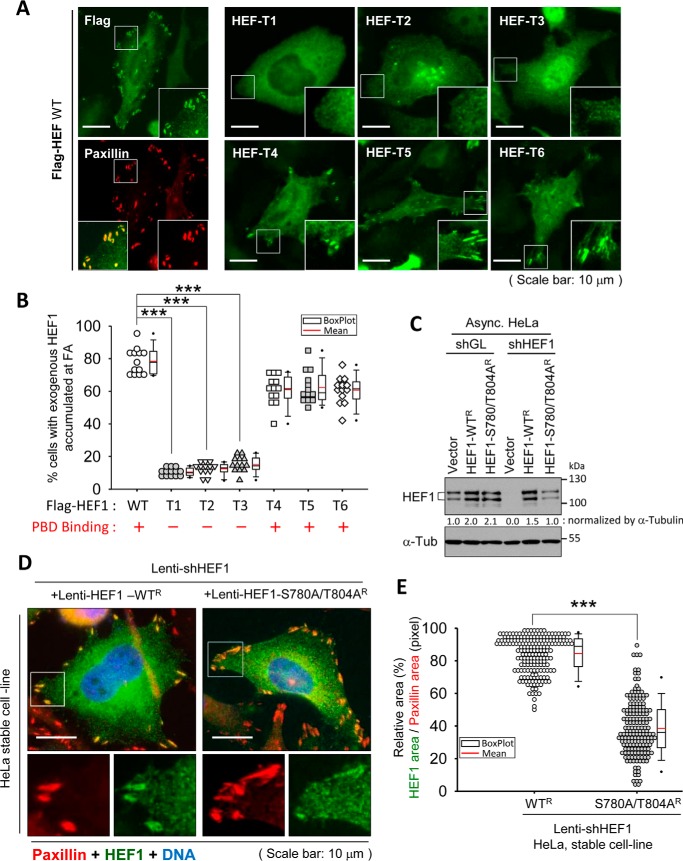
**The HEF1–Plk1 complex induces the correct localization of HEF1 to the FA.** *A* and *B*, the C terminus of HEF1 is required for FA targeting of HEF1 protein. HeLa cells were transfected with FLAG-HEF WT or with each of the truncation mutants (*HEF T1-T6*). Cells were then immunostained with anti-FLAG (*green*) and anti-paxillin (*red*) antibodies. The *enlarged images* in the *bottom left* and on the *right sides* of each image represent the merged and the paxillin (*red*)/FLAG-HEF1 (*green*) signals, respectively (*A*). *Scale bar*, 10 μm. Paxillin was used as the focal adhesion marker. For quantification (*B*), the number of cells with accumulated FLAG (*green*) signal in FA was counted. More than 200 cells were counted in each of the three independent experiments. The percentage of cells with exogenous HEF1 focal adhesion localization is indicated. Each *symbol* represents a percentage in more than 30 cells. The graph shows a combination plot with a dot density plot and a box plot for each category. ***, *p* < 0.001 (one-way ANOVA). Plk1 PBD-binding results (see Fig. 1*D*) are also summarized *below* the graph (*red*). *C*, expression level of HeLa stable cell line. HeLa cells stably expressing the indicated constructs were generated through lentiviral infection. The resulting stably overexpressig cells were infected with either a control luciferase (shGL) or an HEF1-knockdown (shHEF1) lentivirus. The cells were harvested and subjected to immunoblotting. *R*, shHEF1-resistant (3-base mismatch to the shHEF1 sequence). *Vector*, *pHR′-CMV-SV-puro* empty vector. *D*, phosphorylation of Ser-780 and Thr-804 residues on HEF1 induced the FA localization of HEF1. Stably expressing HeLa cells were generated as indicated in *C*. Cells were subjected to an immunostaining assay. *Enlarged images* in the *bottom left* and on the *right sides* of each image represent paxillin (*red*) and HEF1 (*green*) signals, respectively. DNA was stained with DAPI (*blue*). *Scale bar*, 10 μm. *R*, shHEF1-resistant (3-base mismatch to the shHEF1 sequence). Paxillin was used as the focal adhesion marker. *E*, quantification of the relative area of accumulated HEF1 signals at the FA region compared with paxillin signals. The relative area (%) = accumulated HEF1 (*green*) signal at the FA region (pixels)/accumulated paxillin (*red*) signal at the FA region (pixels) × 100. Area was measured using ImageJ. ***, *p* < 0.001 (unpaired two-tailed *t* test). Approximately 200 regions were measured in each of the three independent experiments. Each *symbol* represents an average of the percentages for the three regions. The graph shows a combination plot with a dot density plot and a box plot for each category. All representative images of immunoblot and immunofluorescence were obtained from three independent experiments. Band intensities were quantified with ImageJ and normalized as indicated in the figure, and the relative values are shown *below* the bands.

Because HEF1 is considered to act during FA disassembly (3, 4, 27–29), we monitored the subcellular localizations of HEF1-truncated mutant proteins while focusing on the FA area to investigate whether Plk1 contributes to the FA localization of HEF1. Interestingly, consistent with the PBD pulldown assay, only the C-terminal region–containing HEF1 mutants (T4, T5, and T6), and not the N-terminal constructs (T1, T2, and T3), accumulated at FAs in transfected HeLa cells (Fig. 5, *A* and *B*). FA accumulation of FLAG-HEF1 WT or its C-terminal region-containing HEF1 mutants (T4, T5, and T6) was observed in ∼80 or 60% of transfected HeLa cells, respectively. On the other hand, FA accumulation of HEF1 N-terminal region–containing mutants N terminus-containing mutants was observed only in ∼10% of transfected HeLa cells.

In this experiment, in the presence of endogenous HEF1, overexpression of HEF1 WT occupied nearly 100% (97 ± 9.8% (S.D.)) of the paxillin-stained area. Therefore, to monitor the direct effects of exogenous genes while avoiding the effects of endogenous genes, we generated HeLa cells lacking endogenous HEF1 but stably expressing shRNA-resistant (3-base mismatch to the shHEF1 sequence; see “Experimental procedures” for details) HEF1 WT (HEF1-WT^R^) or its S780A/T804A mutant (HEF1-S780A/T804A^R^) (Fig. 5*C*). As expected, HEF WT accumulated at FAs, covering 85% of the paxillin (FA marker)-stained area. By contrast, HEF1 S780A/T804A mutant did not accumulate at FAs efficiently, covering only 43% (Fig. 5, *D* and *E*). There is less recruitment of the mutant to FAs, and thus the area of staining was reduced. These data suggest the possibility that the phosphorylation of Ser-780 and Thr-804 residues on the HEF1-generated HEF1–Plk1 complex may induce the correct localization of HEF1 to FAs. Notably, a mimetic form of a negatively charged phosphate group from either the Ser-780 residue or the Thr-804 residue (*i.e.* D/E mutant) failed to increase the HEF1–Plk1 PBD interaction (Fig. S1*B*). This suggests the pivotal role of the phosphorylated serine or threonine residue in Plk1 PBD binding, as reported previously (19, 30–32). However, to determine whether these two sites are the sole factor in HEF1 translocation, further experiments should be carried out in the future.

### HEF1–Plk1 complex promotes cell migration

**Figure 6.**
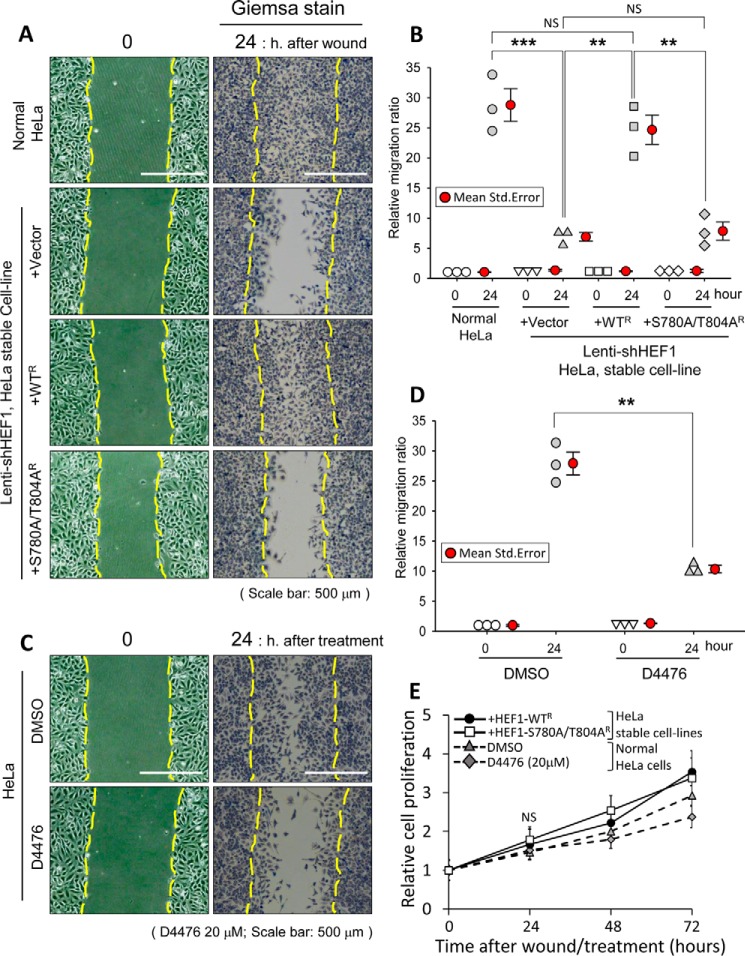
**The HEF1–Plk1 complex promotes cell migration in HeLa cells.** *A* and *B*, the HEF1–Plk1 complex induces cell migration. The same stable cell lines as in Fig. 5 (*D* and *E*) were grown as monolayers until they achieved confluence. Cells were then scratched and photographed immediately (0 h) and after 24 h (24 h). Samples at 24 h were stained with a Giemsa solution (*A*). *Scale bar*, 500 μm. Cells were counted within the *dotted lines* of each photograph, and the relative migration ratio was calculated as follows (*B*). Relative migration ratio = cell number within the *dotted lines* at each time point/each cell number within the *dotted lines* at the time of 0 h. +*Vector*, *pHR′-CMV-SV-puro* empty vector. *R*, shHEF1-resistant (3-base mismatch to the shHEF1 sequence). The graph shows a dot density plot with mean and S.E. (*error bars*) for each category. Each *symbol* represents the average of each independent experiment. All representative images of the wound-healing assay were obtained from three independent experiments. ***, *p* < 0.001; **, *p* < 0.01; *NS*, not statistically significant (one-way ANOVA). *C* and *D*, inhibition of CK1δ activity by treatment with D4476 (33) causes a migration defect of HeLa cells. Asynchronously growing HeLa cells were grown as monolayers and then scratched and treated with either control DMSO or 20 μm D4476 immediately (0 h). Samples at 24 h were stained with a Giemsa solution. Cells were counted within the *dotted lines* of each photograph (*scale bar*, 500 μm), and the relative migration ratio was calculated (*D*) as follows. Relative migration ratio = the cell number within the *dotted lines* at each time point/cell number within the *dotted lines* at the time of 0 h. The graph shows a dot density plot with mean and S.E. (*error bars*) for each category. Each *symbol* represents the average of each independent experiment. All representative images of the wound-healing assay were obtained from three independent experiments. **, *p* < 0.01 (unpaired two-tailed *t* test). *E*, cell proliferation assay was performed using HEF1 stably expressing cell lines and normal HeLa cells. For the same stable cell lines as in *A* (*solid lines*) and HeLa cells under the same conditions as in *C* (*dotted lines*), cell proliferation was measured using an MTT assay. All MTT assays were performed triplicate. The data are the average of three independent experiments. *Error bars*, S.D. *NS*, not statistically significant (unpaired two-tailed *t* test).

Because HEF1 is considered to act during FA turnover (3, 4, 27–29), cancer cell migration activity was monitored using HeLa cells lacking endogenous HEF1 but stably expressing exogenous HEF1 WT or its S780A/T804A mutant, as described in Fig. 5*C*. At 24 h after scratch wounding, the number of WT HeLa cells that migrated into the scratch wound area increased 30-fold compared with that at 0 h. As expected, cells lacking endogenous HEF1, but expressing empty vector (+*Vector*), showed a severe impairment of cell migration. In contrast, the expression of exogenous HEF1 WT (+*WT^R^*) efficiently rescued the cell migration defect associated with the depletion of endogenous HEF1 (+*Vector*), whereas that of a Plk1 binding–defective HEF1 mutant (+*S780A/T804A^R^*) failed to rescue this defect (Fig. 6, *A* and *B*). In this experiment, there was no significant difference in cell proliferation between +WT^R^ and +S780A/T804A^R^ cells (Fig. 6*E*, *solid lines*). These findings further support the pivotal role of the HEF1–Plk1 complex in cell migration.

Because CK1δ was identified as a kinase that phosphorylates Ser-780 and Thr-804 residues of HEF1 in our study (Fig. 4), we next investigated whether cancer cell migration is dependent upon CK1δ activity. The results revealed a severe impairment in cell migration upon the pharmaceutical inhibition of CK1δ by D4476 treatment (33) (Fig. 6, *C* and *D*). In a wound-healing assay, the number of HeLa cells that migrated into the scratched area at 24 h after scratching increased 30-fold in the DMSO control compared with the number at 0 h, whereas cells treated with a CK1δ inhibitor, D4476, showed a less than 10-fold increase in cell migration compared with the sample at 0 h (Fig. 6, *C* and *D*). In addition, treatment of HeLa cells with D4476 (20 μm) for 24 h did not significantly affect cell proliferation, compared with controls treated with DMSO alone (Fig. 6*E*, *dotted lines*). In this experiment, we have found that CK1δ can play a key role in mediating the HEF1–Plk1 interaction but that more needs to be done to determine whether CK1δ is the sole or primary activating kinase in a physiological context.

## Discussion

HEF1 is a scaffold protein, the up-regulation of which promotes FA disassembly and induces cell migration and invasion (3, 4, 27–29). To date, HEF1 has been considered a major factor in cancer cell metastasis (1, 6). This study revealed a new insight into the molecular mechanism underlying the transport of HEF1 to FAs. Here, we demonstrate that HEF1 phosphorylation at Ser-780 and Thr-804 residues by CK1δ induces the HEF1–Plk1 complex formation. This cascade of events ultimately leads to the docking of HEF1 on FAs, and finally, having arrived, HEF1 may exert its FA disassembly activity, leading to cell migration.

Plk1 is a well-known mitotic kinase, whose function appears to be critical for correct M-phase progression (7–9). Here, we showed that, in addition to the role of Plk1 during mitosis, it may also play a surprising role in the early stages of the cell cycle. As events in cell migration occur mostly before mitosis, and as HEF1 forms a complex with Plk1 throughout the cell cycle (Fig. 1, *A* and *B*), the HEF1–Plk1 functional complex required for HEF1 travel might be assembled in the early stages of the cell cycle. These mechanisms reflect the non-mitotic function of Plk1, and although a relatively small amount of Plk1 exists during the S phase compared with that in the M phase, the quantity is sufficient for Plk1 to participate in this very important cellular process during the non-mitotic phase. However, as the HEF1–Plk1 complex and Plk1-dependent mobility shift of the HEF1 protein were observed during mitosis in our experiment (Fig. 1 (*A* and *B*) and Fig. S2), we cannot rule out the possibility that the HEF1–Plk1 complex also functions mitotically. Strong positive correlations between the metastasis of human cancer and the up-regulation of Plk1 have been reported (12, 14); however, none of these studies demonstrated the exact functional mechanism by which Plk1 is involved in metastasis. Our findings reveal the role of Plk1 in metastasis and may provide an answer regarding the functional mechanism involved. We suggest that the docking of Plk1 on HEF1 is essential for HEF1 to translocate to FAs. Therefore, the docking of Plk1 on HEF1 may be a key step in Plk1-induced cell migration or metastasis. The catalytic domains of CK1δ and CK1ϵ are known to be highly conserved (97% identity) (15, 16). Recently, however, it has been demonstrated that there are functional differences between these two kinases (18, 19). In line with this observation, we also evaluated the apparent functional differences between these two kinases in the present study. The activation of CK1δ resulted in the phosphorylation of Ser-780 and Thr-804 residues on HEF1, which serve as Plk1-binding phospho-sites, whereas CK1ϵ did not show any effect on this process. One possible explanation for the functional difference between these two similar kinases is that their HEF1-binding modes may be determined by either their variable N- or C-terminal regions in the pathway, which generate pSer-780 and pThr-804 on HEF1.

In this study, Plk1 directly formed a complex with HEF1, whereas our previous findings showed that Plk1 binds to Dvl2 and then recruits Smad3, an HEF1 degradation component, and hence, HEF1 is dissociated from this destruction complex (19). Therefore, we would like to suggest a dual role of Plk1 in the cellular function of HEF1. One role is the direct regulation through the Plk1-HEF1 complex, resulting in cell migration; the other is its indirect effect through the Plk1-Dvl2 complex, resulting in primary cilium disassembly. These features may arise from the different binding partners in different cell cycle stages: the S phase for cell migration and the G_0_-G_1_ phase for primary cilium disassembly. Our findings should provide new mechanical insights and novel pharmaceutical target sites (HEF1 Ser-780 and Thr-804 residues) regarding the process of HEF1/Plk1-induced metastasis.

## Experimental procedures

### Plasmid construction and mutagenesis

The human, WT form of *HEF1*, or various mutant forms (each a truncation mutant) and an alanine substitution mutant were subcloned into the KpnI-NotI site of *pcDNA-FLAG* (Invitrogen), the EcoRI-NotI site of *pGEX-4T-2* (Amersham Biosciences), or the SalI site of *pHR*′*-CMV-SV-puro* (a gift from Chou-Zen Giam, Uniformed Services University of the Health Sciences, Bethesda, MD). Each full-length and truncation fragment was generated by PCR, using the *pCMV-FLAG-HEF1* WT (a gift from Joel Raingeaud, INSERM, France) as a template. The subcloning of mouse *CK1*δ or *CK1*ϵ was performed as described previously (19). To generate the *HEF1*, shRNA-expressing lentiviral constructs (sh2030), the annealed nucleotides 2030–2048 from the human *HEF1* (accession no. NM-006403) open reading frame (5′-CCGG**CAAAGCCCGTGGAGAATGA**GCTAGC**TCATTCTCCACGGGCTTTG**TTTTTG-3′ (forward) and 5′-AATTCAAAAA**CAAAGCCCGTGGAGAATGA**GCTAGC**TCATTCTCCACGGGCTTTG**-3′ (reverse); the targeting sequences (34) are indicated in boldface type) were subcloned into the AgeI-EcoRI site of the *pLKO.1-puro* vector (a gift of S. A. Stewart and P. A. Sharp, Massachusetts Institute of Technology, Cambridge, MA).

The mutant alleles that are resistant against the silencing effect of the *HEF1* sh2030 RNA, contain three silent mutations (5′-CAAAGCC**A**GT**C**GA**A**AATGA-3′; the three silent mutations are indicated in boldface type). Every mutant was generated using PCR-based site-directed mutagenesis. The target sequence of shGL (CGTACGCGGAATACTTCGA (35)) was subcloned into *pLKO.1-puro* vector in the same manner as shHEF1. Every lentivirus-based shRNA-expressing construct was generated in the same manner to produce *HEF1* shRNA 2030, as described above.

### Cell culture, synchronization, drug treatment, and transfection

HEK293T cells and HeLa cervical carcinoma cell line CCL2 were cultured in 10% FBS containing DMEM, according to the recommendations of the ATCC (Manassas, VA). For the thymidine or nocodazole arrest, HeLa cells were treated with 2.5 mm thymidine (Sigma) or with 200 ng/ml nocodazole (Sigma) for 18 h. Plasmids were transfected to corresponding cells by using Lipofectamine 2000 (Invitrogen), in accordance with the manufacturer's instructions.

### Lentivirus generation and infection

To generate the shRNA-expressing lentiviruses, the *pLKO.1-puro-shLuciferase* (shGL) or -shHEF1 construct was co-transfected with *pHR*′*-CMV-VSV-G* (protein G of vesicular stomatitis virus) and with *pHR*′*-CMV*Δ*R8.2*Δ*vpr* into HEK293T cells. To generate the protein-expressing lentiviruses, the *pHR*′*- CMV-SV-puro-vector-HEF1 wild-type^R^* (where R represents sh2030-resistant silent mutant) *or S780A/T804A^R^* construct was co-transfected with *pHR*′*-CMV-VSV-G* and with *pHR′-CMV*Δ*R8.2*Δ*vpr* into HEK293T cells. To deplete the target genes, HeLa cells were infected with the lentiviruses for 1 day, and then the cells were selected with 4 μg/ml puromycin for 2–3 days. To generate an expression-knockdown stable cell line, the cells were first infected with the expression viruses, and then the knockdown viruses were infected. To avoid the side effects of protein tags, lentiviral expression constructs do not contain protein tags.

### Immunoprecipitation, immunoblotting, and mass spectrometry analysis

For immunoprecipitation, the cells were harvested with 1× ice-cold PBS and then lysed with 1× TBSN buffer (20 mm Tris-Cl (pH 8.0), 150 mm NaCl, 0.5% Nonidet P-40, 5 mm EGTA, 1.5 mm EDTA, 10 mg/ml *p*-nitrophenyl phosphate (Sigma), and a protease inhibitor mixture (Roche, Mannheim, Germany)). The cell lysates were then centrifuged at 15,000 × *g* for 20 min at 4 °C. The resulting clear lysates were incubated with the indicated antibodies for 4–6 h at 4 °C and were then incubated with protein A- or G-Sepharose beads (Santa Cruz Biotechnology, Inc., Dallas, TX) for an additional 3 h at 4 °C. The beads were then washed with 1× TBSN more than four times, and then 2× Laemmli sample buffer (4% SDS, 20% glycerol, 120 mm Tris-Cl (pH 6.8), 10% 2-mercaptoethanol, and 0.02% bromphenol blue) was added. The precipitated beads were then boiled for 10 min at 95–100 °C and then subjected to immunoblotting analyses.

Immunoblotting was carried out as follows. Samples were separated by SDS-PAGE and then transferred to a PVDF membrane. The membranes were incubated with primary antibodies for 2 h at room temperature (or overnight at 4 °C) and with HRP-conjugated secondary antibodies (Amersham Biosciences) for 1 h at room temperature. After extensive washing with 1× TBST (50 mm Tris-Cl (pH 7.5), 150 mm NaCl, 0.05% Tween 20), the immunoreactive signals were detected through exposure to a radiographic film, using an enhanced chemiluminescence (ECL) detection system (Pierce). Antibodies used in this study are listed in Table S1.

To identify the *in vivo* phosphosites, HEK293T cells were transfected with wild-type *pFLAG-HEF1* and then treated with 2.5 mm thymidine (Sigma) or with 200 ng/ml nocodazole (Sigma) for 18 h, to arrest the cells in the S or M phase. The resulting cells were subjected to IP with an anti-FLAG antibody (Sigma) and precipitated using protein G-agarose beads. The samples were separated by 10% SDS-PAGE and stained with the GelCode Blue Stain Reagent (Pierce), according to the manufacturer's instructions. The FLAG-HEF1 band was excised from the gel, to detect the HEF1 *in vivo* phosphosites. The excised gels were digested in-gel with trypsin (Promega) and then used in the mass spectrometry analyses as described previously (19). The peptides were separated by LC-MS/MS or nanoLC-MS/MS/MS on an LTQ-Orbitrap XL or LTQ linear ion trap mass spectrometer (Thermo Electron Corp., San Jose, CA). To identify the phosphopeptides and determine the sequences from MS/MS and MS/MS/MS data, SEQUEST (Thermo Electron Corp.) software was used.

### GST-PBD–binding and peptide-binding assays

The *GST-PBD* WT and the *GST-PBD* phosphopincer mutant (AM; H538A/K540M) (a gift from Michael B. Yaffe, Massachusetts Institute of Technology) were expressed in *Escherichia coli BL21* (DE3) through isopropyl-β-d-1-thiogalactopyranoside (IPTG) induction and purified with GSH-agarose. The bead-conjugated *GST-PBD* WT or AM was incubated with a clear cell lysate for 1 h at 4 °C and then precipitated by brief centrifugation. The precipitated beads were washed with 1× TBSN more than four times and boiled in 2× Laemmli sample buffer. The samples were then subjected to immunoblotting analyses. For the peptide-binding assay, peptides were conjugated with beads using the SulfoLink coupling gel system (Pierce), according to the manufacturer's instruction. The bead-conjugated peptides were incubated with clear cell lysates for 2 h at 4 °C, and then the same steps used in the GST-PBD pulldown assay were followed, as described above.

### Protein expression and purification for crystallization

The Polo box domain of mouse Plk1 (Swiss Prot entry Q07832, residues 367–603; indicated in Fig. 3*A*) was expressed as a recombinant protein containing an N-terminal His_6_ tag in the pET28a vector and a tobacco etch virus protease cleavage site (sequences ENLYFQS, where the amino acid residue between Q and S is cleaved) was engineered between the affinity tag and Plk1 PBD. Recombinant protein was expressed and purified following the procedure described earlier (25). Briefly, the protein was expressed in *E. coli* BL21 (DE3) CodonPlus RIL (Stratagene) at 18 °C; the expression was induced by 0.5 mm IPTG, and the cells were cultured for 16 h. The cells were harvested and lysed in 25 mm HEPES (pH 7.5), 300 mm NaCl, 5 mm β-mercaptoethanol, and 0.1 mm PMSF by sonication and centrifugation. The supernatant after centrifugation was applied to an Ni^2+^-nitrilotriacetic acid affinity chromatography column (GE Healthcare) and eluted with a linear gradient of 20–500 mm imidazole in 25 mm HEPES (pH 7.5), 300 mm NaCl, 5 mm β-mercaptoethanol followed by digestion with tobacco etch virus protease (1:10 molar ratio) overnight. It was further purified by a HiLoad 26/600 Superdex-75 gel filtration column (GE Healthcare) pre-equilibrated with 25 mm HEPES (pH 7.5), 300 mm NaCl, 5 mm β-mercaptoethanol, and purified proteins were treated with 10% glycerol. The phosphopeptides, pSer-780 and pThr-804, corresponding to ^775^KVMNSpSNQLC^784^ and ^799^LHYPSpTTALQE^809^ of HEF1 were synthesized with Ser-780 and Thr-804 phosphorylated.

### Crystallization, data collection, structure solution, and refinement

Purified Plk1 PBD was concentrated to 10 mg/ml before complexation, and the pSer-780 and pThr-804 peptides were added in a 1:3 molar ratio and incubated overnight. Initial crystallizations were performed using the sitting-drop vapor-diffusion method at 295 K using a robotics system mosquito (TTP Labtech). The diffraction quality crystals were obtained only for the Plk1 PBD and pThr-804 peptide complex by mixing an equal volume of the complex in 25 mm HEPES (pH 7.5), 300 mm NaCl, 5 mm β-mercaptoethanol with the reservoir solution consisting of 0.1 m sodium citrate, pH 5.5, 15% (v/v) PEG 6000. Needle crystals appeared in a few days. The crystals were cryoprotected using reservoir solution supplemented with an addition of 30% (*v/v*) ethylene glycol and were flash-cooled in liquid nitrogen. X-ray diffraction data were collected at 100 K on beamline 5C equipped with an ADSC Quantum 315r CCD detector at Pohang Light Source (Pohang, Korea). The crystal belonged to space group *P*2_1_, with unit-cell parameters *a* = 57.564, *b* = 59.440, *c* = 72.748 Å, α = γ = 90°, β = 99.48°. The crystal diffracted X-ray to 2.9 Å Bragg spacings, and the completeness was less than 85%. X-ray diffraction data were processed and scaled using the HKL2000 program (36). The Matthews coefficient was 2.51 Å^3^ Da^−1^, and the estimated solvent content was 51.0%, resulting in two Plk1 PBD molecules in an asymmetric unit. The structure was solved by molecular replacement using the known structure as a search model (PDB code 3HIK using only the protein atoms) with the program PHENIX (37). The resulting electron density map with Plk1 PBD revealed clear density for the bound peptide. Because the terminal residues of the peptide were not clearly visible in the electron density maps, they were left out in the final model. Manual building was performed using *Coot* (38), and the final crystallographic refinement was performed using the program PHENIX (39). Seven water molecules that were clearly defined in the electron density maps were included in the final model. The final model was validated using PROCHECK (40). Solvent-accessible and interaction areas were calculated by PISA (http://www.ebi.ac.uk/msd-srv/prot_int/pistart.html),^6^ and figures were generated using PyMOL (41). The statistics of data collection and refinement are summarized in Table 2.

### Antibody production

The rabbit polyclonal anti-phosphoantibodies were raised against the HEF1 pSer-780 and pThr-804 epitopes by using synthetic peptides NH_2_-KVMNSpSNQLC-COOH (aa 775–784) and NH_2_-LHYPSpTTALQE-COOH (aa 799–809), respectively (AbFRONTIER Inc., Seoul, Korea), and then purified by affinity chromatography. The production of anti-phosphoantibodies was monitored by an ELISA in every bleeding serum. To improve the specificity of the anti-phosphoantibody, 5 μg/ml non-phosphorylated peptide was added to each antibody solution.

### In vitro kinase assays

The *GST-HEF1* WT, the *GST-HEF1* alanine substitution mutants, the *GST-CK1*δ-WT, and the *GST-CK1*δ kinase-inactive mutant (K38M) were expressed in *E. coli* BL21 (DE3) through IPTG induction and were then purified with GSH-agarose (Sigma). The kinases and putative substrates were mixed in a kinase mixture (50 mm Tris-Cl (pH 7.5), 10 mm MgCl_2_, 5 mm dithiothreitol, 2 mm EGTA, 10 mg/ml *p*-nitrophenyl phosphate (Sigma), and 1X protease inhibitor mixture (Roche)) in the presence of 10 μm cold-ATP and 10 μCi of [γ-^32^P]ATP for 30 min at 30 °C. The reaction was stopped by the addition of 2× Laemmli sample buffer and then boiled for 10 min at 95 °C. The samples were separated by SDS-PAGE. The gels were stained with Coomassie Brilliant Blue (CBB) and dried. The autoradiogram signal was detected through radiography. To determine incorporated phosphate, the time course of GST-HEF1 WT and GST-HEF1 S780A/T804A mutant phosphorylation was measured at 0 h, 30 min, 1 h, and 2 h by liquid scintillation counting of dried SDS-polyacrylamide gel bands. For liquid scintillation counting, either GST-HEF1 WT or GST-HEF1 S780A/T804A mutant band was excised from dried SDS-polyacrylamide gel band, and then gel pieces were dissolved in 30% H_2_O_2_ (Sigma) at 50 °C for 4 h. After heating at 37 °C for an additional 1 h to drive off residual O_2_, a scintillation mixture (ULTIMA GOLD, PerkinElmer Life Sciences) was added to sample, and the [γ-^32^P]radioactivity was measured using a liquid scintillation analyzer (Tri-Carb 2910 TR, PerkinElmer Life Sciences).

### Immunofluorescence assay and image analyses

The cells were grown on the coverslips and fixed with 4% paraformaldehyde for 10 min at room temperature and then permeabilized with ice-cold pure methanol for 2 min. The resulting cells were subjected to indirect immunofluorescence analyses. The primary antibodies were incubated for 2 h at room temperature (or overnight at 4 °C) and washed with 1× PBST (1× PBS + 0.1% Triton X-100) more than four times. An Alexa Fluor 488 (green)- or a Texas Red (red)-conjugated secondary antibody (Invitrogen) was incubated for 1 h at room temperature after washes with 1× PBST following incubation with the primary antibody. The DNA was stained with 0.1 μg/ml DAPI (Sigma) solution and incubated for 10 min at room temperature. The resulting coverslips were mounted on the glass slides by using a Fluoro-Gel mounting medium (EMS, Hatfield, PA). The resulting samples were observed and photographed through either a Zeiss LSM 700 confocal microscope or a Nikon Eclipse ti-u inverted fluorescence microscope system. The antibodies used in the immunofluorescence analyses were as follows: rabbit anti-paxillin (Abcam; 1:200), mouse anti-FLAG (Sigma; 1:100), mouse anti-vinculin (Abcam; 1:200), and mouse anti-HEF1 (Cell Signaling; 1:100).

To measure the area of the FA, images were acquired either using the Zeiss LSM 700 confocal microscope at 1024 × 1024 pixels and 16-bit resolution or the Nikon Eclipse ti-u inverted fluorescence microscope at 1280 × 1024 pixels and 16-bit resolution and then analyzed with either the ImageJ or the MetaMorph imaging software (Molecular Devices, Sunnyvale, CA). The area of the HEF1 and paxillin signals (pixels) in the HEF1-paxillin overlap region was measured by ImageJ. Then the relative area was calculated as follows. The relative area (%) = accumulated HEF1 (green) signal at the FA region (pixels)/accumulated paxillin (red) signal at the FA region (pixels) × 100.

### Wound-healing assay

For the wound-healing assay, the cells were plated on 12-well tissue culture plates and grown until they became confluent. The confluent cells were scratched with sterilized pipette tips and then washed with sterilized 1× PBS. The culture plates were marked along the scratch region immediately after scratching. The scratch-wounded cells were replenished with either normal complete medium or inhibitor-containing medium and then photographed at the indicated time point. After 24 h of wounding, the cells were stained with a Giemsa solution (Sigma), according to the manufacturer's instructions. The cells were photographed using a Nikon Eclipse ti-u inverted microscope at a ×40 magnification. Cells were counted within the *dotted lines* of each photograph in a similar number of all 0 h cells. The number of cells at 0 h is in the range of 16–19 cells.

### MTT cell proliferation assay

Cell proliferation was performed using the Cyto X cell viability assay kit (LPS Solution Corp., Daejeon, Korea) according to the manufacturer's instructions. Briefly, cells were seeded into 96-well microplates at a density of 2 × 10^3^ cells/well and incubated for 24 h at 37 °C in 5% CO_2_ condition. Cell viability was measured after 0, 24, 48, and 72 h. Cells were treated with 10 μl of Cyto X reagent and incubated for an additional 1 h at 37 °C in 5% CO_2_ condition. The absorbance was measured using an ELISA plate reader (SPECTRA MAX 190, Molecular Devices, Sunnyvale, CA) at a wavelength of 450 nm.

### Statistical analysis

Statistical analyses were performed using unpaired two-tailed *t* test for Figs. 5*E* and 6*D* and one-way ANOVA for Figs. 5*B* and 6*B*.

## Author contributions

K. H. L. and E. E. K. designed the experiments; K. H. L., J.-A. H., S.-O. K., N.-K. S., J.-H. J., S.-K. K., J. H. K., S. C. S., J. K. B., K. R., and B. Y. K. performed the experiments; K. H. L., J.-A. H., S.-O. K., K. S. L., K. R., B. H. J., H. C.-M., H. G. L., Y. T. K., J. S. A., E. E. K., and B. Y. K. analyzed the data; K. H. L., E. E. K., and Y. T. K. wrote the paper. All authors reviewed the results and approved the final version of the manuscript.

## Supplementary Material

Supporting Information

## Footnotes

6 Please note that the JBC is not responsible for the long-term archiving and maintenance of this site or any other third party hosted site.

## Footnotes

5 The abbreviations used are:

FA: focal adhesion
FAK: focal adhesion kinase
PBD: Polo box domain
CK1: casein kinase 1
aa: amino acids
IP: immunoprecipitation
IPTC: isopropyl 1-thio-β-d-galactopyranoside
PDB: Protein Data Bank
CBB: Coomassie Brilliant Blue
ANOVA: analysis of variance
MTT: 3-(4,5-dimethylthiazol-2-yl)-2,5-diphenyltetrazolium bromide.
