# Supplementary material for: Phosphorylation of human enhancer filamentation 1 (HEF1) stimulates interaction with Polo-like kinase 1 leading to HEF1 localization to focal adhesions
Source: J Biol Chem. 2017 Nov 30;293(3):847–62. doi: 10.1074/jbc.M117.802587 (PMC5777258; doi:10.1074/jbc.M117.802587)
Supplement: Supporting Information [file supp_293_3_847__index.html]

Phosphorylation of human enhancer filamentation 1 (HEF1) stimulates interaction with Polo-like kinase 1 leading to HEF1 localization to focal adhesions — Phosphorylation of human enhancer filamentation 1 (HEF1) stimulates interaction with Polo-like kinase 1 leading to HEF1 localization to focal adhesions — HEF1 translocation and cell migration by HEF1–Plk1 complex — Supporting Information 

# Phosphorylation of human enhancer filamentation 1 (HEF1) stimulates interaction with Polo-like kinase 1 leading to HEF1 localization to focal adhesions

## Supporting Information

- Supplemental Data (.docx, 376 KB) - Supplemental Figure, Figure Legend, and Table
