# Supplementary material for: Phosphorylation of human enhancer filamentation 1 (HEF1) stimulates interaction with Polo-like kinase 1 leading to HEF1 localization to focal adhesions
Source: J Biol Chem. 2017 Nov 30;293(3):847–62. doi: 10.1074/jbc.M117.802587 (PMC5777258; doi:10.1074/jbc.M117.802587)
Supplement: Supporting Information [file 10.1074_M117.802587_jbc.M117.802587-1.docx]

**SUPPLEMENTAL INFORMATION**

**Phosphorylation of human enhancer filamentation 1 (HEF1) stimulates interaction with Polo-like kinase 1 leading to HEF1 localization to focal adhesions**

Kyung Ho Lee*, Jeong-Ah Hwang, Sun-Ok Kim, Jung Hee Kim, Sang Chul Shin, Eunice EunKyeong Kim, Kyung S. Lee, Kunsoo Rhee, Byeong Hwa Jeon, Jeong Kyu Bang, Hyunjoo Cha-Molstad, Nak-Kyun Soung, Jae-Hyuk Jang, Sung-Kyun Ko, Hee Gu Lee, Jong Seog Ahn, Yong Tae Kwon*, and Bo Yeon Kim*

*** Corresponding author. E.mail: [leekh@kribb.re.kr](mailto:leekh@kribb.re.kr), yok5@snu.ac.kr, [bykim@kribb.re.kr](mailto:bykim@kribb.re.kr)

**SUPPLEMENTAL FIGURE LEGENDS**

**Supplementary Figure S1.** (**A**) Both HEF1 p-S780 and p-T804 peptides were identified by mass spectrometry analysis. HEK293T cells transfected with Flag-tagged HEF1 WT were treated with either 2.5 mM thymidine or 200 ng/ml nocodazole for 18 h, and the resulting cells were harvested and subjected to an immunoprecipitation assay with an anti-Flag antibody. The precipitates were separated using 10 % SDS-PAGE and stained Flag-HEF1 bands were excised from the gel (**A**) and subsequently analyzed by mass spectrometry to identify the *in vivo* phospho-sites (Table 1). Peptides identified in the mass spectrometry analyses are displayed in Table 1.

(B) Substitution of S780 or T804 with a negatively charged residue caused a reduction in HEF1-Plk1 PBD interaction. Either the Flag-tagged HEF1 T6-WT or each of the D/E substitution mutants (“S” to “D/E” or “T” to “D/E”) was transfected into HEK293T cells. The resulting cell lysates were subjected to PBD pull-down assays using GST-Plk1 PBD WT, and then membranes were immunoblotted with an anti-Flag antibody. CBB represents the amount of loaded GST-Plk1 PBD WT protein. All representative images of immunoblot were obtained from three independent experiments. Band intensities were quantified with ImageJ and normalized as indicated in the figure, and the relative values are shown below the bands.

**Supplementary Figure S2. Plk1-dependent mobility shift of HEF1 protein during cell cycle progression.** HeLa cells were depleted using either control luciferase (shGL) or Plk1 (shPlk1) by shRNA-expressing lentiviral infection. The resulting cells were synchronized via a double thymidine block and released. Cells were then harvested at the indicated time points and subjected to immunoblotting. G_1_/S, G_1_/S-phase. M, mitotic phase. Noc, nocodazole. Representative images of immunoblot were obtained from two independent experiments. Band intensities were quantified with ImageJ, and the relative values are shown below the bands.

**Supplementary Table S1. List of antibodies used in this study.**

**
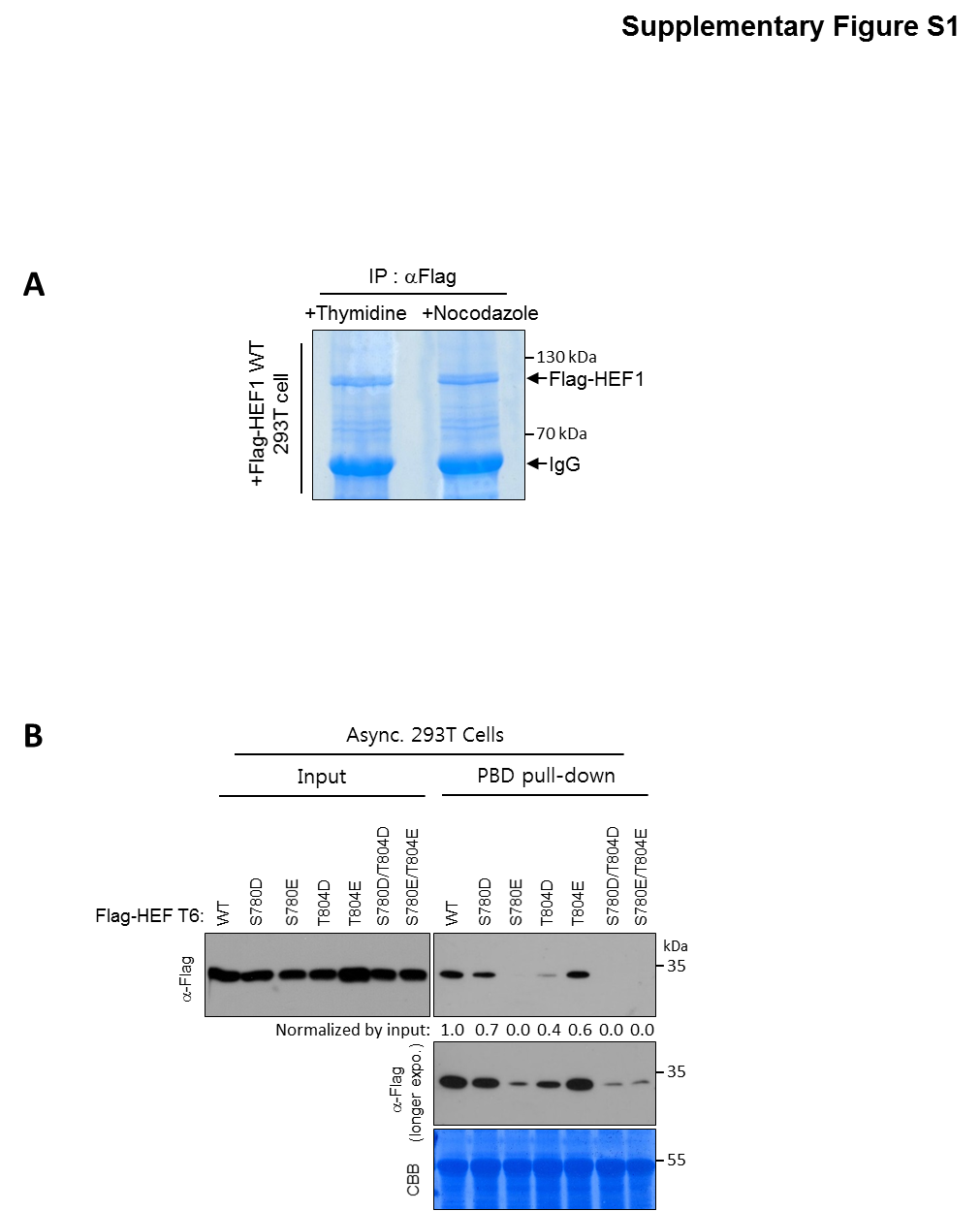
**

**
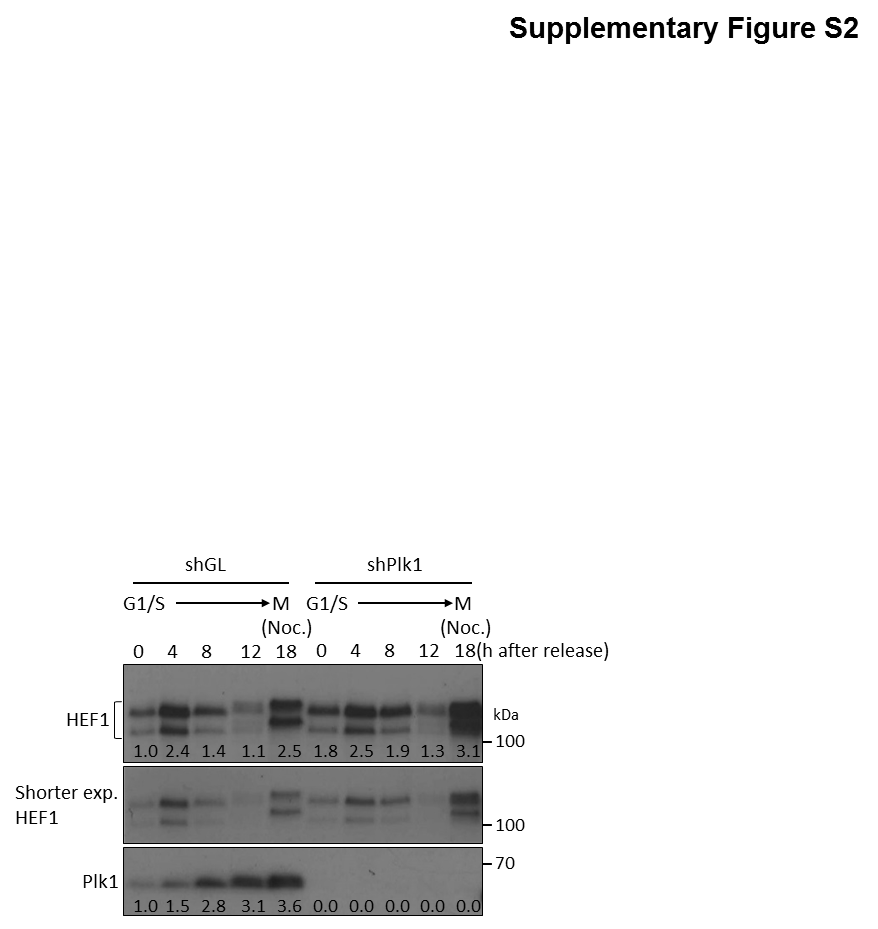
**

**
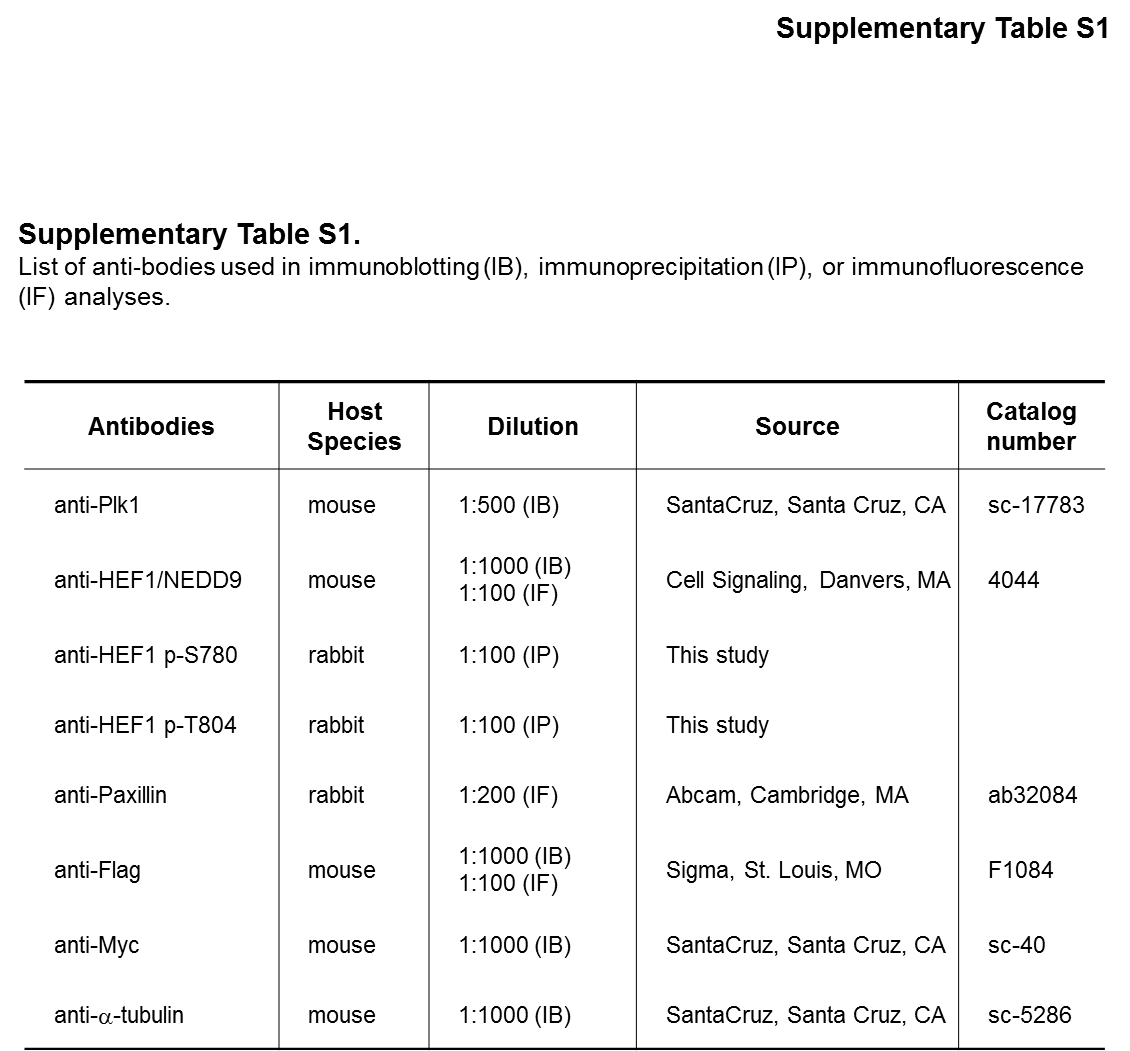
**
